# An Italian adaptation of the Child-Adolescent Perfectionism Scale: Testing measurement invariance across grade levels and exploring associations with academic achievement

**DOI:** 10.1371/journal.pone.0255814

**Published:** 2021-08-06

**Authors:** Michele Vecchione, Mariacarolina Vacca

**Affiliations:** 1 Department of Social and Developmental Psychology, Sapienza University of Rome, Rome, Italy; 2 Department of Psychology, Sapienza University of Rome, Rome, Italy; Aalborg University, DENMARK

## Abstract

This study aims to examine the properties of an Italian version of the Child-Adolescent Perfectionism Scale (CAPS), one of the most widely used instrument for the assessment of self-oriented (SOP) and socially-prescribed (SPP) perfectionism in young people. The study was conducted on two large samples of middle (*n* = 379, M_age_ = 11.31) and high school (*n* = 451, M_age_ = 15.21) students. Confirmatory factor analysis supported the expected three-factor structure, comprising SOP-Striving, SOP-Critical, and SPP. Multigroup analyses provided evidence of configural, metric, and (partial) scalar measurement invariance across grade levels. Structural invariance (i.e., the invariance of factor variances and covariances) was also established. The scale scores exhibited a differentiated pattern of relations with personality traits and academic achievement, as measured by school grades: SOP-Critical and SPP were positively related to neuroticism and have adverse effects on grades of middle and high school students, respectively. SOP-Striving, by contrast, was positively related to conscientiousness and predicted higher grades. The SOP-Striving-achievement relation was consistent across grade levels and held even after controlling for individual differences in conscientiousness and neuroticism. In sum, results from this study establish sound psychometric properties for an Italian version of the CAPS, providing support for the dual nature of self-oriented perfectionism among adolescents of different ages.

## Introduction

Perfectionism is a multifaceted construct encompassing both personal and social aspects [1]. While different conceptualizations of the constituent dimensions have been provided [2–4] the tripartite model by Hewitt and Flett [5] is one of the most established. The model differentiates between *self-oriented* perfectionism (i.e., the beliefs that being perfect and striving for perfection are important), *socially prescribed* perfectionism (i.e., the belief that others expect perfection from oneself, and that their acceptance is conditional on meeting these expectations), and *other-oriented* perfectionism (i.e., a tendency to set unrealistic standards for others).

The differential effects of these forms of perfectionism on the individual’s functioning has been investigated in a number of studies [6]. Results have shown that Socially Prescribed Perfectionism (SPP) is related to poor psychological adjustment and an increased vulnerability to health problems [7, 8]. Consequently, it was conceived as encompassing the maladaptive side of perfectionism, what is called perfectionistic concerns [9]. Self-Oriented Perfectionism (SOP) is related to a number of beneficial outcomes–including goal attainment [10] and problem-focused coping [11]–and only to a minor extent to dysfunctional characteristics [12]. It was accordingly regarded as an indicator of adaptive perfectionism, referred to as perfectionistic strivings [13, 14]. Other-oriented perfectionism (OOP) represents a distinct aspect, which does not overlap with the above dimensions and showed unique associations with antisocial and narcissistic personality trait [15, 16].

Most of these findings derive from samples of adults [6]. Yet, there is a growing interest and research on the potential effects of perfectionism at earlier developmental stages [17–22]. Authors generally agree that one of key periods for the development of this trait is adolescence [13, 23, 24]. This might be related to the profound changes that occur during this transitional period [25], along with the increasing demands from school, which encourage adolescents’ unrealistic expectations and self-critical tendencies [26], especially in light of their heightened sensitivity to the influences of the social environment [24].

The scientific interest for perfectionism in adolescents has been fueled by the acknowledgment of its clinical implications [27]. Systematic evidence has shown that perfectionistic concerns can be particularly detrimental to children and adolescents mental health [28]. For example, SPP in adolescence has been linked to anxiety and depression [24], self-harm [29], negative affect [30], and difficulties in emotional regulation [31].

### The Child-Adolescents Perfectionism Scale: Measuring perfectionism at earliest ages

One of the most extensively used measure of perfectionism in children and adolescents is the Child-Adolescents Perfectionism Scale (CAPS, [32, 33]). The CAPS is an adaptation of the Multidimensional Perfectionism Scale (MPS), developed by the same authors for the assessment of perfectionism in adults [5]. In its original form [32, 33], the CAPS consisted of 22 items, seven of which are negatively keyed, intended to measure SOP and SPP. Differently from the MPS, OOP was not included, as it appears to play a marginal role in adolescence [32, 33].

The scale has been translated in several languages and used in a number of studies with children and adolescents aged 8 and over (for a recent and comprehensive review, see Vicent, Rubio-Aparicio, Sánchez-Meca & Gonzálvez [34]). Subsequent factor-analytic studies, however, have questioned the original two-factor structure of the CAPS. In a first study, McCreary, Joiner, Schmidt and Ialongo [35] examined the dimensionality of the scale in a sample of 11- and 12-year-old African American students, using exploratory factor analysis. Results have shown that a three-factor solution, with SOP split into two facets, provided a better representation of the data. The two SOP factors encompass a tendency for overly critical self-evaluations, that is accompanied by distress over failure (labelled as SOP-Critical–e.g., “I get mad at myself when I make a mistake”), and a tendency to set high standards of performance, without associated criticism (labelled as SOP-Striving–e.g., “I try to be perfect in everything I do”). Eight items with weak loadings, including the seven negatively keyed items, have been removed, resulting in a revised 14-item version. In a later study, O’Connor, Dixon and Rasmussen [36] compared the original two-factor structure to McCreary et al. [35] three-factor model, using confirmatory factor analysis on data from two samples of 15- and 16-year-old Canadian students. Results provided support for the three factor-structure. Similar to the McCreary et al. [35] study, only 14 positively keyed items were retained, although the two versions did not entirely overlap.

Empirical findings supported the importance of separating the two SOP factors, which exhibited a differentiated pattern of associations with relevant outcomes [35–40]. SOP-Critical has been predominantly related to maladaptive outcomes, while SOP-Striving has showed significant associations with functional characteristics, especially when controlling for the overlap with SOP-Critical [1]. This is consistent with the distinction between positive strivings and maladaptive evaluative concerns that has been addressed in the adult literature [1, 41].

### The present study

In line with the above theoretical framework, this study aims to develop and validate an Italian version of the CAPS in two large samples of middle and high school students. We decided to use the version proposed by O’Connor et al. [36] (henceforth referred to as CAPS-14) for several reasons: (a) it is more parsimonious compared to the original, 22-item version; (b) the proposed three-factor structure is consistent with recent evidence about the dual nature of the SOP in children and adolescents; (c) it does not include negatively-keyed items. Despite the use of reversed items may have some merits [42], it represents a controversial procedure that can be especially problematic in research with children [43].

We first examined the psychometric properties of the CAPS-14 in terms of dimensionality and internal consistency. We expect to replicate the three-factor structure observed in earlier studies [35, 36]. The goodness of fit of the proposed model was compared with that of a two-factor model including SOP as a unitary factor, as originally conceptualized for the CAPS [32, 33].

We then investigated the measurement and structural invariance of the scale across middle and high school students. The CAPS was developed for use in children and adolescent populations, but little is known about its equivalence across different ages and grade levels. As it has been noticed by O’Connor et al. [36] in the validation study of the CAPS-14, it would be important to establish whether the selected items are core to the measurement of child and adolescent perfectionism, and whether they are invariant across age. To the best of our knowledge, this is the first attempt to shed light on this issue.

Construct validity was investigated by calculating Pearson correlations of the CAPS-14 scales with conscientiousness and neuroticism. Among the Big Five, these are the traits most strongly and consistently related to perfectionism [44]. Meta-analytic evidence suggests that perfectionistic striving (e.g., SOP) was related to high conscientiousness, whereas perfectionistic concern (e.g., SPP) was related to high neuroticism [45]. These findings have been found in adults [46] as well as in children [47] and adolescents [44]. To the best of our knowledge, however, no studies have used the CAPS to investigate how the two SOP factors relate to these personality dimensions. This is relevant especially considering that, among the Big Five, conscientiousness and neuroticism are the traits mostly related to important life outcomes [48]. Based on the conceptual meaning of the CAPS scales, and in accordance with extant literature, we expect SOP-Striving to be positively related to conscientiousness, and SOP-Critical and SPP to be positively related to Neuroticism.

Criterion validity was investigated by examining associations of the CAPS-14 scales with students’ academic achievement, as measured by school grades. In this regard, a recent meta-analysis [49] has shown that perfectionistic strivings were related to high academic achievement (GPA, grades, or exam performance) in samples of different ages. Perfectionistic concern, by contrast, had a negative relation to academic achievement, especially when the overlap with perfectionistic strivings is controlled for. This relation, however, was generally weak and not always significant across studies [50].

To date, only three studies have examined the relationship between academic achievement and perfectionism by using the CAPS on samples of children and adolescents. Two of them [22, 51] have adopted the original two-factor model [32, 33] yielding inconsistent findings. Stornelli, Flett and Hewitt [22] have found that SOP and SPP were mostly unrelated to achievement test scores in a sample of elementary school students. Damian, Stoeber, Negru-Subtirica and Băban [51] reported a positive association of self-reported GPA with SOP, and negative but non-significant associations with SPP in a sample of high school students. The study by Harvey, Moore and Koestner [38] was the only study to examine the differential impact of the two SOP facets on academic achievement. Findings from a sample of elementary school children showed positive associations between SOP-striving and school grades. The associations with SOP-Critical was non-significant, while SSP was not included in the study.

The present study adds to the literature by examining the joint contribution of SOP-Critical, SOP-Striving and SPP to school grades at different ages (i.e., among middle and high school students). Moreover, it investigates whether the CAPS scales have incremental validity with respect to conscientiousness and emotional stability, which are *important correlates of* both perfectionism [52] and academic achievement [53]. This adds to our understanding of whether the relationship with school grades is unique to a specific dimension of perfectionism or reflects common variance with broader personality traits.

## Materials and method

### Participants and procedure

Participants were 830 children and adolescents recruited from two Italian public schools, one middle and one high school. Of these, 379 were middle school students aged 10 to 13 (47% female), with a mean of 11.31 (SD = .64). The other 451 participants were high school students aged 14 to 18 (37% female), with a mean of 15.21 (SD = 1.01). At the beginning of the second semester (February), students completed a self-report questionnaire including the CAPS-14 and measures of conscientiousness and emotional stability, along with other instruments that are not relevant to this study. The questionnaire was administered during classroom hours by two trained psychologists, under the supervision of a teacher. The research was presented as aiming at examining personality correlates of academic engagement. Participation was voluntary and was requested only from students whose parents had given their informed consent. All of the students were assured of the confidentiality of their responses. The study was approved by the ethical committee of the Department of Psychology (Sapienza University of Rome).

### Measures

*The Child-Adolescent Perfectionism Scale (CAPS)*. Participants completed an Italian version of the CAPS-14 [36]. The scale includes fourteen items divided into three subscales: SPP, SOP-Critical and SOP-Striving. Each item was rated on a 5-point Likert scale ranging from 1 (not at all true of me) to 5 (very true of me). A forward translation from the original U.S. version into Italian was performed independently by a professional translator and the first author. The two forward translations were then compared and combined into a single version. A back-translation into the original language was performed by an Italian native speaker who was fluent in English. The original and back-translated versions were then compared and checked for consistency and clarity [54]. The final Italian version of the CAPS-14 is reported in Table 2.

*Conscientiousness and Emotional stability*. Middle school students completed 12 items taken from a shortened, 30-item version of the Big Five Questionnaire for Children (BFQ-C), an instrument for the assessment of the Big-5 personality factors in children [55]. The items used in this study are taken from the conscientiousness (e.g., “I do things with much care and attention”) and emotional stability (e.g., “I easily get angry”) subscales of the BFQ-C. Each item was rated on a 5-point Likert scale ranging from 1 “Almost never true” to 5 “Almost always true”. In the present sample, Cronbach’s alpha reliability coefficients were .74 for conscientiousness, and .82 for emotional stability.

High school students completed the conscientiousness and neuroticism subscales of a shortened version of the Big Five Inventory (BFI-S) [56] (see Ubbiali, Chiorri, Hampton & Donati [57], for an Italian adaptation of the full-length BFI). The two subscales include three items each. Examples are: “does things efficiently” (conscientiousness), and “worries a lot” (neuroticism). For each item, participants were asked to indicate the extent to which they agree with the statement, using a 5-point Likert scale ranging from “strongly disagree” to “strongly agree.” In the present sample, alpha reliability coefficients were .70 for conscientiousness, and .60 for neuroticism.

*Academic achievement*. Students’ grades were obtained from school records at the end of the scholastic year (June). In both groups, grades were assigned by the teachers on a scale that ranges from 1 to 10, in accordance with the European educational system. A composite measure of academic achievement was created by averaging grades of different subject matters (e.g., mathematics, Italian, history, foreign languages).

## Results

### Descriptive statistics

Table 1 reports descriptive statistics, Cronbach’s alpha reliability coefficients, and intercorrelations between the CAPS-14 subscales.

**Table 1:** Descriptive statistics and scale intercorrelations for the CAPS-14.

	Middle school			High school		
*Scales*	1.	2.	3.	1.	2.	3.
1. SOP–Striving		.45	.48		.57	.39
2. SOP–Critical			.48			.49
3. SPP						
*M*	3.25	2.68	3.08	3.02	2.56	2.76
*SD*	1.04	.91	.78	.91	.76	.75
Skewness	-.25	.21	-.33	-.10	.15	-.11
Kurtosis	-.63	-.52	-.10	-.43	-.12	-.26
Cronbach’s Alpha	.71	.65	.76	.71	.65	.81

SOP: self-oriented perfectionism; SPP: socially prescribed perfectionism.

Alphas ranged from .65 (SOP–Striving) to .76 (SPP) in the middle school group, and from .65 (SOP–Striving) to .81 (SPP) in the high school group. Given the well-known limitation of alpha as an index of internal consistency [58], in the subsequent analyses reported below we further examined reliability by means of McDonald’s [59] omega coefficient. Omega is a model based-reliability coefficient that has less strict assumptions than alpha, representing a more appropriate approach to the assessment of internal consistency [60].

### Structural validity

The internal validity of the CAPS-14 was performed by means of Confirmatory Factor Analysis (CFA). The proposed three-factor structure of the CAPS-14 was tested against the data obtained in both groups of middle and high school students. Model parameters were estimated using the maximum likelihood robust (MLR) estimation procedure available in Mplus 6.1 [61]. For each factor, the loading and intercept of a single item (i.e., the reference indicator) were fixed to 1 and 0, respectively. This allows to identify the model and set the scale of latent factors. The choice of the reference indicators may have important implications for subsequent tests of invariance. Several studies have indeed shown that using items that are not invariant across groups could lead to misleading results [62, 63]. The best possible invariant indicators were therefore identified by adopting the *Minχ*^*2*^ approach, as recommended by Thompson, Song, Shi & Liu [64]. We first fitted a baseline model, in which factor loadings and intercepts were all constrained to be equal across groups. Then, we tested a set of models in which the parameters of a single item were freely estimated, whereas the rest remained constrained. The item that produced the smallest increase in the chi-square statistic with respect to the baseline model was selected as reference indicator of the respective factor. Model fit was assessed with chi-square statistics, the Comparative Fit Index (CFI), the Root Mean Square Errors of Approximation (RMSEA), and the Standardized Root Mean Square Residuals (SRMR).

A competing model was tested and compared with the expected three-factor model, using the Yuan and Bentler scaled chi-squared difference test (ΔYB-χ^2^) [65, 66]. This model posited SOP and SPP as two correlated factors, without differentiating between the SOP facets, as in the original version of the CAPS [32, 33].

The three-factor model fits adequately in both middle, χ^2^(74) = 150.50, *p* < .001, CFI = .928, RMSEA = .053, (.040, .065), SRMR = .052, and high school groups, χ^2^(74) = 217.94, *p* < .001, CFI = .906, RMSEA = .066, (.056, .076), SRMR = .053. Factor loadings, intercorrelations among latent factors, and their internal consistency (omega coefficients), are reported in Table 2.

**Table 2:** A CFA on the CAPS-14: Standardized factor loadings, correlations between latent factors, and composite reliability.

	Middle school			High school		
*Items*	F1: SOP–Striving	F2: SOP–Critical	F3: SPP	F1: SOP–Striving	F2: SOP–Critical	F3: SPP
2. I want to be the best at everything I do	.66			.71		
**4. I always try for the top score on a test**	.66			.59		
14. I try to be perfect in everything I do	.69			.71		
6. I get mad at myself when I make a mistake		.40			.37	
8. I can’t stand to be less than perfect		.71			.71	
**10. I get upset if there is even one mistake in my work**		.62			.69	
12. Even when I pass, I feel that I have failed if I didn’t get one of the highest marks in the class		.53			.51	
1. My teachers expect my work to be perfect			.49			.42
3. There are people in my life who expect me to be perfect			.71			.73
5. My family expects me to be perfect			.67			.68
**7. Other people always expect me to be perfect**			.77			.82
9. People expect more from me than I am able to give.			.58			.52
11. Other people think I have failed if I do not do my very best all the time			.37			.51
13. I feel that people ask too much of me			.36			.61
Factor correlations and omega reliability coefficients	1.	2.	3.	1.	2.	3.
1. SOP–Striving	.*71*			.*71*		
2. SOP–Critical	.67	.*66*		.84	.*66*	
3. SPP	.66	.62	.*77*	.51	.65	.*81*

* *p* < .05

*p* ** < .01. The reference indicators are shown in bold. Reliability coefficients (McDonald’s omega) are reported in italics on the main diagonal of the correlation matrix.

Standardized factor loadings ranged from .36 to .77 in the middle school group, and from .37 to .82 in the high school group. T*he three subscales* were positively related, with correlations in the range from .51 (SOP-Striving–SPP, high school) to .84 (SOP-Striving–SOP-Critical, high school). Omegas reliability coefficients ranged from .66 (SOP-Critical) to .77 (SPP) in the middle school group, and from .66 (SOP-Critical) to .81 (SPP) in the high school group.

The two-factor model exhibited a less than ideal fit in both samples: middle school, χ^2^(76) = 194.76, *p* < .001, CFI = .888, RMSEA = .065, (.053, .076), SRMR = .058; high school, χ^2^(76) = 243.60, *p* < .001, CFI = .890, RMSEA = .070, (.060, .080), SRMR = .057. The chi-square was significantly worse when compared with the three-factor model (middle school: ΔYB-χ^2^ (2) = 40.77, *p* < .001; high school: ΔYB-χ^2^ (2) = 24.49, *p* < .001). This provides statistical support for the discriminant validity of the two SOP facets at different ages.

### Measurement invariance across grade levels

The measurement and structural equivalence of the CAPS-14 across grade levels was investigated through a multigroup CFA of mean and covariance structures [67]. We assessed measurement invariance at a set of increasingly restricted levels. In Model 1 (configural invariance), the same three-factor model was fitted in both middle and high school samples, without imposing any between-group constraint. This level of invariance tested the equality of the overall structure (i.e., the same number of factors and the same pattern of loadings) across groups. In Model 2 (metric invariance), the factor loadings were constrained to be equal across groups. This level of invariance is required to establish that the measurement unit is the same in each group. In Model 3 (scalar invariance), additional equality constraints on item intercepts were imposed. This level of invariance is required to establish that scores have the same origin in each group. Finally, we investigated the invariance of structural parameters, by constraining the variances and covariances of the latent factors to be equal across groups. If full metric, scalar or structural invariance were not supported, we tested for partial invariance [68], by releasing the constraints on the items that turned out not to be invariant.

We adopted multiple criteria for comparing the fit of these nested, *increasingly restricted* models. We calculated the Yuan-Bentler chi-square difference test, with an alpha level of .05. Nevertheless, this test has the same limitations as the chi-square test for assessing overall fit [69]. We therefore additionally considered differences in McDonald’s Non-Centrality Index (MNCI), RMSEA, and SRMR between constrained and unconstrained models [70, 71]. According to Kang et al.’s [70], a change larger than .010 in the MNCI would indicate non-invariance. Chen [71] suggested a criterion of .015 for the RMSEA, while the cut-off values for the SRMR were set to .030 and .010 for metric and scalar invariance, respectively. Although the change in CFI was one of the most adopted approach for the assessment of measurement invariance in past studies, its use has been recently discouraged [70]. It was therefore not considered as criterion in the present study. Results of measurement invariance tests were reported in Table 3.

**Table 3:** Tests of measurement invariance across grade levels.

	Model fit				
	YB-χ^2^	df	RMSEA	SRMR	MNCI
1. Configural	368.47**	148	.060	.053	.875
2. Metric	392.93**	159	.060	.060	.868
3. Scalar	555.21**	170	.074	.067	.792
4. Partial scalar ^a^	416.31**	165	.061	.059	.859
5. Factor variances and covariances	434.44**	171	.061	.064	.852
	Model comparison				
	ΔYB-χ^2^	Δdf	ΔRMSEA	ΔSRMR	ΔMNCI
2 vs. 1	23.88*	11	.000	.007	-.007
3 vs. 2	186.52**	11	.014	.007	-.076
4 vs. 2	24.50**	6	.001	-.001	-.009
5 vs. 4	18.36**	6	.000	.005	-.006

*Note*.

* *p* < .05

*p* ** < .01.

^a^ Five intercepts were not invariant across groups: 3 for SPP (items 5, 9, and 11) and 1 each for SOP-STR (item 2) and SOP-CRI (item 6).

The configural model (Model 1) fit the data, suggesting that the same structure holds in each group. Constraining the loadings across groups (Model 2) led to minor changes in the MNCI (-.007) and the SRMR (.007), while the RMSEA remained unchanged. Hence, the hypothesis of full metric invariance appears supported by the data. Constraining the intercepts (Model 3) led to non-negligible changes in the MNCI (-.076). This suggests that the equivalence of intercepts is not completely tenable. Inspection of modification indices revealed that 5 equality constraints contributed most to the lack of invariance. We therefore compared Model 2 with a model that had the additional constraint of 6 intercepts, whereas the remaining 5 were free to vary (Model 4). The differences between the two models in the MCNI (-.009), the RMSEA (.001), and the SRMR (-.001) met the recommended criteria. The assumption of partial scalar invariance can be therefore retained. The variances and covariances of the latent factors also appeared to be equivalent, as the decrement in fit that was observed after these parameters were constrained to equality turned out to be not insubstantial.

### Associations with the conscientiousness and neuroticism personality traits

Table 4 reports zero-order Pearson’s correlations of the CAPS-14 with conscientiousness and neuroticism.

**Table 4:** Correlations between the CAPS-14 scales and personality measures.

	Middle school			High school		
	SOP–Striving	SOP–Critical	SPP	SOP–Striving	SOP–Critical	SPP
Conscientiousness	.25** (.25**)	.06 (-.11*)	.13*(.00)	.43** (.40**)	.18**(-.07)	.13**(-.02)
Neuroticism	.13** (-.10*)	.24** (.11*)	.28** (.20**)	.01 (-.13*)	.17** (.13**)	.22*(.18**)

* *p* < .05

** *p* < .01. Coefficients outside the parenthesis represent bivariate (zero-order) correlations. Coefficients in parentheses are partial correlations controlling for the variance common to the three CAPS-14 subscales.

All correlations were positive and statistically significant, except for the ones between conscientiousness and SOP–Critical, and between neuroticism and SOP–Striving. As expected, conscientiousness was mostly related to SOP–Striving, whereas neuroticism was mostly related to SOP-Critical and SSP. Partial correlations, controlling for the overlap among the CAPS-14 subscales are shown in the parentheses of Table 4. We found that conscientiousness correlated positively with SOP-Striving, but showed null or weak negative correlations with SOP-Critical and SPP. The opposite pattern was observed for neuroticism, which correlated negatively with SOP-Striving, and positively with SOP-Critical and SPP.

### Associations with academic achievement

The criterion validity of the Italian adaptation of the CAPS-14 was assessed by examining its relationship with students’ final grades. A hierarchical regression was conducted in both middle and high school students. The composite scores of the three CAPS scales were entered into the regression at step 1. Composite scores of conscientiousness and neuroticism were entered at step 2. This allows to investigate whether perfectionism predict grades after basic personality disposition were partialled out. Results are shown in Table 5, which includes regression coefficients of each predictor at the step in which it was entered, along with change in R-squared.

**Table 5:** Standardized regression weights and proportion of variance accounted in scholastic achievement.

	Middle school	High school
SOP–Striving	.23**	.24**
SOP–Critical	-.19*	-.01
SPP	-.11	-.15**
*R*^*2*^	.04	.05
SOP–Striving	.17*	.13*
SOP–Critical	-.15*	.01
SPP	-.08	-.14**
Conscientiousness	.12*	.25**
Neuroticism	-.11	.00
*ΔR*^*2*^	.03	.05
*R*^*2*^	.07	.10

* *p* < .05

** *p* < .01.

At the first step, SOP-Striving predicted higher grades in both groups. SOP-Critical and SSP, by contrast, predicted grades negatively among middle and high school students, respectively. At step 2, conscientiousness was significantly related to higher grades in both groups. The unique contribution of neuroticism was not significant. After the two traits were controlled for, the effects of the CAPS-14 scales observed at the first step remained statistically significant. Taken together, the three CAPS-14 subscales and the two basic personality traits accounted for 7% and 10% of the variance in grades among middle and high school groups, respectively.

## Discussion

The current study aimed to develop and validate an Italian version of the CAPS-14, a self-report scale for the assessment of self- and other-oriented perfectionism [36]. Confirmatory factor analysis supported the expected three-factor structure (comprising SOP-Striving, SOP-Critical and SPP), in two samples of middle and high school Italian students. Factor loadings were mostly adequate, averaging .59 (*SD* = .14) and .61 (*SD* = .13) in middle and high school groups, respectively. There were, however, few items, such as items 11 and 13 in the middle school group, and item 6 in the high school group, that loaded less than .40 on the respective factor, thus demonstrating a weaker convergent validity.

The scale scores showed acceptable to good levels of internal consistency for SOP-Striving and SPP. Coefficients for SOP-Critical, by contrast, were lower than .70, the most widely used threshold for appropriate reliability [72]. It is well acknowledged, however, that cut-off criteria should be used with caution [73]. To further investigate the internal consistency of SOP-Critical, we calculated the inter-item correlations among the four items of the subscale. Correlations ranged from .26 to .50 in the middle school group (*M* = .40), and from .13 to .49 in the high school group (*M* = 32). This represents more than adequate levels of item homogeneity [74].

As expected, the three factors were highly correlated. All r’s were in the range of .50 to .67, with a notable exception being the latent correlation of .84 between SOP-Striving and SOP-Critical among high school students. The two SOP facets correlated less strongly (*r* = .67), in the middle school sample. This finding might be due to the developmental characteristics of examined samples, covering distinct sub-stages of adolescence [75] namely early adolescence (i.e., the middle school sample, aged 10 to 13) and middle adolescence (i.e., the high school sample, aged 14 to 18). According to the Social Expectations Model [24], parental expectations and criticism play a central role in the emergence of perfectionism. During early adolescence, perceived criticism from others is a primary source of perfectionism [76]. With the development of self-determination [77], middle adolescents may have internalized external expectations and associated self-evaluation [24]. Thus, striving for perfectionism (SOP-Striving) and self-criticism (SOP-Critical) might be more closely intertwined at this age. It should be noted, however, as discussed later in the manuscript, that constraining the correlations among the CAPS-14 factors to be equal across school levels did not result in a substantial deterioration of model fit. Thus, although descriptively higher among high school students, the correlation between SOP-Striving and SOP-Critical can be considered as equivalent across groups. Additional research efforts are needed to deepen our understanding of whether and how the overlap between SOP-Striving and SOP-Critical progressively change as children go through different stages of development.

As a next step, we investigated the measurement invariance of the scale across school levels. Findings provided evidence of configural and full metric invariance across middle and high school groups. In other words, the items of the CAPS-14 were related to the three factors in the same way and with the same strength in both groups. When scalar invariance was tested, we found that five items (3 from the SPP scale) had non invariant intercepts across grade levels. A partial invariance model, in which some invariance restrictions are relaxed, was tested and found to be statistically defensible. We acknowledged that the procedure we used to test for partial scalar invariance (i.e., relaxing constraints iteratively, until adequate fit is achieved) is subjected to inflated risk of capitalizing on chance [78]. It is encouraging, however, that 9 out of 14 items, including the reference indicators (64% of the total) have both equivalent loadings and intercepts across groups. Thus, partial scalar invariance appears to be a reasonable assumption. Structural invariance was also established. When constrained to equality across groups, both variances and covariances among latent factors were found to be invariant. Hence, correlations among latent factors can also be regarded as invariant [68]. If confirmed, these findings suggest that the fourteen items of the CAPS can be invariably used to assess perfectionism at different ages across adolescence.

Construct validity was supported by correlations with measures of conscientiousness and neuroticism personality traits. As expected, SOP-Striving was positively related to conscientiousness, whereas SOP-Critical and SPP were positively related to neuroticism. This correlation pattern was consistent across grade levels and turned out to be clearer once the overlapping variance between the three CAPS scale scores was controlled. In this regard, research suggests that partialling techniques are essential to understand the complex nature of the relationship of multidimensional measures of perfectionism with indicators of psychological adjustment and maladjustment [79].

We also investigated the associations between students’ scores on the CAPS-14 and academic achievement, as measured by school grades. We found that SOP-Striving uniquely predicted grades. That is, striving for perfection led to better learning outcomes, once the impact of self-criticism and beliefs about other expectations was removed. Of interest, the predictive validity of SOP-Striving was consistent across middle and high-school grade levels, and remained significant after controlling for conscientiousness and neuroticism. The effect of this form of self-oriented perfectionism was therefore not attributable to the variance it shares with conscientiousness, a closely related trait that is consistently related to academic achievement (for conceptual distinctions between adaptive perfectionism and conscientiousness [80]. This accords with earlier research findings [81], showing that perfectionism is able to predict academic engagement, over and above the effects of conscientiousness and neuroticism.

Furthermore, SPP and SOP-Critical exhibited detrimental effects on academic achievement, though differently across the two samples. SPP significantly predicted lower grades among high school students, but not among middle school sample. This might be due to the increased vulnerability to others’ expectations that is observed during middle adolescence [51]. On the other hand, SOP-Critical was negatively related to grades in the middle school sample, while did not contribute significantly in the high school sample. Thus, self-criticism appears to exert a more significant role among youngest. Future studies are needed to unravel the mechanisms through which socially prescribed and self-critical perfectionism adversely affect academic achievement at different ages.

The present study has some limitations. First, the scale was translated into Italian, and we did not have the opportunity to test the equivalence with the original, English form [32]. Future studies should assess the measurement invariance of the scale across languages. A further limitation of the study is the exclusive reliance on self-reported data for the assessment of perfectionism. Such data are subject to socially desirable responding and other response biases, including halo effect, with the associated risk of inflated correlations among variables. The use of multiple informants can provide additional, unique information about correlates of SOP-Striving, SOP-Critical, and SPP. Moreover, it can provide more valid estimates of the overlap among the three domains of perfectionism. An additional potential drawback is the use of different trait measures in middle and high school student samples. This precluded the possibility of testing whether the relationship of the CAPS-14 with conscientiousness and neuroticism was statistically equivalent and thus generalizable across different age groups. Finally, our investigation was restricted to early and middle adolescence. Widening the age range of participants would allow to enhance our understanding about the functioning of the scale at different stages of the development.

Despite these limitations, results from this study establish sound psychometric properties for an Italian version of the CAPS-14. Given the data at hand, a potential concern might be related to the discriminant validity of the two SOP latent factors, which are highly interrelated in both groups (the constrained latent correlation in the invariant model was .78). However, several considerations must be kept in mind regarding this result. First, the correlation between the two factors was below, although slightly so, the acceptable level of .85 [82, 83]. This is especially important if one considers that the restrictive assumption of zero cross-loadings in the CFA usually leads to over-estimated correlations among latent factors [84]. Second, the three-factor model fitted significantly better with respect to the original two-factor structure. This provides statistical justification for differentiating the two SOP factors. Third, and likely most importantly, the two factors exhibited differential relations with personality traits (i.e., conscientiousness and neuroticism) and important academic outcomes (i.e., school grades). All in all, these findings appear to suggest the importance of separating the two SOP facets, thus supporting the dual nature of self-oriented perfectionism among adolescents of different ages.

## References

[1] Stoeber J, Otto K. Positive conceptions of perfectionism: Approaches, evidence, challenges. *Pers Soc Psychol Rev* 2006; 10: 295–319. doi: 10.1207/s15327957pspr1004_2 17201590

[2] Blankstein KR, Dunkley DM. Evaluative concerns, self-critical, and personal standards perfectionism: A structural equation modeling strategy. In: *Perfectionism*: *Theory*, *research*, *and treatment*. Washington, DC, US: American Psychological Association, 2002, pp. 285–315.

[3] Frost RO, Marten P, Lahart C, et al. The dimensions of perfectionism. *Cognit Ther Res* 1990; 14: 449–468.

[4] Rice KG, Ashby JS, Slaney RB. Self-esteem as a mediator between perfectionism and depression: A structural equations analysis. *J Couns Psychol* 1998; 45: 304–314.

[5] Hewitt P, Flett G. Dimensions of perfectionism in unipolar depression. *J Abnorm Psychol* 1991; 100: 98–101. doi: 10.1037//0021-843x.100.1.98 2005279

[6] Limburg K, Watson HJ, Hagger MS, Egan SJ. The relationship between perfectionism and psychopathology: A meta-analysis. *J Clin Psychol* 2017; 73: 1301–1326. doi: 10.1002/jclp.22435 28026869

[7] Molnar DS, Sadava SW, Flett GL, Colautti J. Perfectionism and health: A mediational analysis of the roles of stress, social support and health-related behaviours. *Psychol Health*; 20. doi: 10.1080/08870446.2011.630466 22149004

[8] *The psychology of perfectionism*: *Theory*, *research*, *applications*. New York, NY, US: Routledge/Taylor & Francis Group, 2018.

[9] Stoeber J, Childs JH. The assessment of self-oriented and socially prescribed perfectionism: Subscales make a difference. *J Pers Assess* 2010; 92: 577–585. doi: 10.1080/00223891.2010.513306 20954059

[10] Powers TA, Koestner R, Topciu RA. Implementation intentions, perfectionism, and goal progress: Perhaps the road to hell is paved with good intentions. *Pers Soc Psychol Bull* 2005; 31: 902–912. doi: 10.1177/0146167204272311 15951362

[11] Dunkley D, Blankstein K, Halsall J, Williams M, Winkworth G. The relation between perfectionism and distress: Hassles, coping, and perceived social support as mediators and moderators. *J Couns Psychol* 2000; 47: 437–453.

[12] Corry J, Green M, Roberts G, Fullerton JM, Schofield PR, Mitchell PB. Does perfectionism in bipolar disorder pedigrees mediate associations between anxiety/stress and mood symptoms? *Int J Bipolar Disord* 2017; 5: 34. doi: 10.1186/s40345-017-0102-8 28983840 PMC5629191

[13] Stoeber J, Childs JH. Perfectionism. In: Levesque RJR, editor. *Encyclopedia of adolescence*. New York: Springer, pp. 2053–2059.

[14] Stoeber J, Feast AR, Hayward JA. Self-oriented and socially prescribed perfectionism: Differential relationships with intrinsic and extrinsic motivation and test anxiety. *Pers Individ Dif* 2009; 47: 423–428.

[15] Sherry SB, Mackinnon SP, Gautreau CM. Perfectionists do not play nicely with others: Expanding the social disconnection model. In: Sirois FM, Molnar DS, editors. *Perfectionism*, *health*, *and well-being*. Cham, Switzerland: Springer International Publishing AG, 2016, pp. 225–243.

[16] Stoeber J. How other-oriented perfectionism differs from self-oriented and socially prescribed perfectionism: Further findings. *J Psychopathol Behav Assess* 2015; 37: 611–623.

[17] Essau CA, Leung PWL, Conradt J, Cheng H, Wong T. Anxiety symptoms in Chinese and German adolescents: their relationship with early learning experiences, perfectionism, and learning motivation. *Depress Anxiety* 2008; 25: 801–810. doi: 10.1002/da.20334 17592617

[18] Freudenstein O, Valevski A, Apter A, Zohar A, Shoval G, Nahshoni E, et al. Perfectionism, narcissism, and depression in suicidal and nonsuicidal adolescent inpatients. *Compr Psychiat* 2012; 53: 746–752. doi: 10.1016/j.comppsych.2011.08.011 22364727

[19] Goodwin H, Haycraft E, Meyer C. Psychological risk factors for compulsive exercise: A longitudinal investigation of adolescent boys and girls. *Pers Individ Dif* 2014; 68: 83–86.

[20] Hewitt PL, Caelian CF, Chen C, Flett GL. Perfectionism, stress, daily hassles, hopelessness, and suicide potential in depressed psychiatric adolescents. *J Psychopathol Behav Assess* 2014; 36: 663–674.

[21] Leone EM, Wade TD. Measuring perfectionism in children: a systematic review of the mental health literature. *Eur Child Adolesc Psychiatry* 2018; 27: 553–567. doi: 10.1007/s00787-017-1078-8 29098468

[22] Stornelli D, Flett GL, Hewitt PL. Perfectionism, achievement, and affect in children: A comparison of students from gifted, arts, and regular programs. *Can J Sch Psychol* 2009; 24: 267–283.

[23] Flett GL, Coulter L-M, Hewitt PL, Nepon T. Perfectionism, rumination, worry, and depressive symptoms in early adolescents. *Can J Sch Psychol* 2011; 26: 159–176.

[24] Flett GL, Hewitt PL, Oliver JM, Macdonald S. Perfectionism in children and their parents: A developmental analysis. In: Flett GL, Hewitt PL, editors. *Perfectionism*: *Theory*, *research*, *and treatment*. Washington, DC, US: American Psychological Association, 2002, pp. 89–132.

[25] Damian LE, Negru-Subtirica O, Pop EI, Baban A. The costs of being the best: Consequences of academic achievement on students’ identity, perfectionism, and vocational development. In: Montgomery A, Kehoe I, editors. *Reimagining the purpose of schools and educational organisations*: Cham: Springer International Publishing, pp. 173–188.

[26] Herman KC, Wang K, Trotter R, Reinke WM, Ialongo N. Developmental trajectories of maladaptive perfectionism among African American adolescents. *Child Dev* 2013; 84: 1633–1650. doi: 10.1111/cdev.12078 23480846 PMC3695018

[27] Gilman R, Ashby JS. A first study of perfectionism and multidimensional life satisfaction among adolescents. *J Early Adolesc* 2003; 23: 218–235.

[28] Morris L, Lomax C. Review: Assessment, development, and treatment of childhood perfectionism: a systematic review. *Child Adolesc Ment Health* 2014; 19: 225–234. doi: 10.1111/camh.12067 32878354

[29] O’Connor RC, Rasmussen S, Hawton K. Predicting depression, anxiety and self-harm in adolescents: The role of perfectionism and acute life stress. *Behav Res Ther* 2010; 48: 52–59. doi: 10.1016/j.brat.2009.09.008 19818954

[30] Damian LE, Stoeber J, Negru O, Băban A. Positive and negative affect in adolescents: an investigation of the 2 × 2 model of perfectionism. *Cogn Brain Behav* 2014; 18: 1–16.

[31] Vois D, Damian LE. Perfectionism and emotion regulation in adolescents: A two-wave longitudinal study. *Pers Individ Diff* 2020; 156: 109756.

[32] Flett GL, Hewitt PL, Boucher DJ, Davidson LA, Munro Y. The child–adolescent perfectionism scale: Development, validation, and association with adjustment. Unpublished manuscript; 1997.

[33] Flett GL, Hewitt PL, Besser A, Su C, Vaillancourt T, Boucher D, et al. The Child-Adolescent Perfectionism Scale: Development, psychometric properties, and associations with stress, distress, and psychiatric symptoms. *J Psychoeduc Assess* 2016; 34: 634–652.

[34] Vicent M, Rubio-Aparicio M, Sánchez-Meca J, Gonzálvez C. A reliability generalization meta-analysis of the child and adolescent perfectionism scale. *J Affect Disord* 2019; 245: 533–544. doi: 10.1016/j.jad.2018.11.049 30445380

[35] McCreary BT, Joiner TE, Schmidt NB, Ialongo NS. The structure and correlates of perfectionism in African American children. *J Clin Child Adolesc Psychol* 2004; 33: 313–324. doi: 10.1207/s15374424jccp3302_13 15136196

[36] O’Connor RC, Dixon D, Rasmussen S. The structure and temporal stability of the Child and Adolescent Perfectionism Scale. *Psychol Assess* 2009; 21: 437–443. doi: 10.1037/a0016264 19719354

[37] Affrunti NW, Woodruff-Borden J. Negative affect and child internalizing symptoms: The mediating role of perfectionism. *Child Psychiatry Hum Dev* 2016; 47: 358–368. doi: 10.1007/s10578-015-0571-x 26215173

[38] Harvey BC, Moore AM, Koestner R. Distinguishing self-oriented perfectionism-striving and self-oriented perfectionism-critical in school-aged children: Divergent patterns of perceived parenting, personal affect and school performance. *Pers Individ Diff* 2017; 113: 136–141.

[39] McArdle S, Duda JL. Exploring the etiology of perfectionism and perceptions of self-worth in young athletes. *Soc Dev* 2008; 17: 980–997.

[40] Soreni N, Streiner D, McCabe R, Bullard C, Swinson R, Greco A, et al. Dimensions of perfectionism in children and adolescents with obsessive-compulsive disorder. *J Can Acad Child Adolesc Psychiatry* 2014; 23: 136–143. 24872829 PMC4032082

[41] Dunkley DM, Blankstein KR, Masheb RM, Grilo CM. Personal standards and evaluative concerns dimensions of “clinical” perfectionism: A reply to Shafran et al. (2002, 2003) and Hewitt et al. (2003). *Behav Res Ther* 2006; 44: 63–84. doi: 10.1016/j.brat.2004.12.004 16301015

[42] Billiet JB, McClendon MJ. Modeling acquiescence in measurement models for two balanced sets of items. *Struct Equ Modeling* 2000; 7: 608–628.

[43] Weijters B, Baumgartner H, Schillewaert N. Reversed item bias: An integrative model. *Psychol Methods* 2013; 18: 320–334. doi: 10.1037/a0032121 23646990

[44] Stoeber J, Otto K, Dalbert C. Perfectionism and the Big Five: Conscientiousness predicts longitudinal increases in self-oriented perfectionism. *Pers Individ Diff* 2009; 47: 363–368.

[45] Stricker J, Buecker S, Schneider M, Preckel F, Kandler C. Multidimensional perfectionism and the Big Five personality traits: A Meta-Analysis. *Eur J Pers* 2019; 33: 176–196.

[46] Rice KG, Ashby JS, Slaney RB. Perfectionism and the Five-Factor Model of personality. [60] Assessment 2007; 14: 385–398. doi: 10.1177/1073191107303217 17986656

[47] Parker WD, Stumpf H. An examination of the Multidimensional Perfectionism Scale with a sample of academically talented children. *J Psychoeduc Assess* 1995; 13(4): 372–383.

[48] Roberts BW, Kuncel NR, Shiner R, Caspi A, and Goldberg LR. The power of personality: the comparative validity of personality traits, socioeconomic status, and cognitive ability for predicting important life outcomes. *Perspect Psychol Sci* 2007; 2: 313–345. doi: 10.1111/j.1745-6916.2007.00047.x 26151971 PMC4499872

[49] Madigan DJ. A meta-analysis of perfectionism and academic achievement. *Educ Psychol Rev* 2019; 31: 967–989.

[50] Stoeber J. Perfectionism and performance. In: Murphy SM, editor. *Oxford Handbook of Sport and Performance Psychology*. New York: Oxford University Press, pp. 294–306.

[51] Damian LE, Stoeber J, Negru-Subtirica O, Băban A. On the development of perfectionism: The longitudinal role of academic achievement and academic efficacy. *J Pers* 2017; 85: 565–577. doi: 10.1111/jopy.12261 27237456

[52] Smith MM, Sherry SB, Vidovic V, Saklofske DH, Stoeber J, Benoit A. Perfectionism and the Five-Factor Model of personality: A meta-analytic review. *Pers Soc Psychol Rev* 2019; 23: 367–390. doi: 10.1177/1088868318814973 30612510

[53] Poropat AE. A meta-analysis of the five-factor model of personality and academic performance. *Psychol Bull* 2009; 135: 322–338. doi: 10.1037/a0014996 19254083

[54] Beaton DE, Bombardier C, Guillemin F, Ferraz MB. Guidelines for the process of cross-cultural adaptation of self-report measures. *Spine* 2000; 25: 3186–3191. doi: 10.1097/00007632-200012150-00014 11124735

[55] Barbaranelli C, Caprara GV, Rabasca A, Pastorelli C. A questionnaire for measuring the Big Five in late childhood. *Pers Individ Diff* 2003; 34: 645–664.

[56] Lang FR, John D, Lüdtke O, Schupp J, Wagner GG. Short assessment of the Big Five: Robust across survey methods except telephone interviewing. *Behav Res* 2011; 43: 548–567. doi: 10.3758/s13428-011-0066-z 21424189 PMC3098347

[57] Ubbiali A, Chiorri C, Hampton P, Donati D. Psychometric properties of the Italian adaptation of the Big Five Inventory (BFI). *Appl Psychol Bull* 2013; 266: 37–48.

[58] Sijtsma K. On the use, the misuse, and the very limited usefulness of Cronbach’s alpha. *Psychometrika* 2009; 74: 107–120. doi: 10.1007/s11336-008-9101-0 20037639 PMC2792363

[59] McDonald RP. *Test theory*: *A unified treatment*. Psychology Press, 2013.

[60] Chen FF, Hayes A, Carver CS, Laurenceau JP, Zhang Z. Modeling general and specific variance in multifaceted constructs: A comparison of the bifactor model to other approaches. *J Pers* 2012; 80: 219–251 doi: 10.1111/j.1467-6494.2011.00739.x 22092195

[61] Muthén LK, Muthén BO. Mplus: Statistical analysis with latent variables–User’s Guide. 2012.

[62] Cheung GW, Rensvold RB. Testing factorial invariance across groups: A reconceptualization and proposed new method. J Manage 1999; 25: 1–27.

[63] Johnson EC, Meade AW, DuVernet AM. The role of referent indicators in tests of measurement invariance. Struct Equ Modeling 2009; 16: 642–657.

[64] Thompson YT, Song H, Shi D, Liu Z. It Matters: Reference Indicator Selection in Measurement Invariance Tests. Educ Psychol Meas 2021; 81: 5–38. doi: 10.1177/0013164420926565 33456060 PMC7797958

[65] Yuan KH, Bentler PM. Three likelihood-based methods for mean and covariance structure analysis with non-normal missing data. Sociol Methodol 2000; 30: 165–200.

[66] Asparouhov T, Muthén B. Robust chi square difference testing with mean and variance adjusted test statistics. *Mplus Web Notes* No. 10 2006.

[67] Meredith W. Measurement invariance, factor analysis and factorial invariance. *Psychometrika* 1993; 58: 525–543.

[68] Byrne BM, Shavelson RJ, Muthén B. Testing for the equivalence of factor covariance and mean structures: The issue of partial measurement invariance. *Psychol Bull* 1989; 105: 456–466.

[69] Steenkamp J-BEM, Baumgartner H. Assessing measurement invariance in cross-national consumer research. *J Consum Res* 1998; 25: 78–90.

[70] Kang Y, McNeish DM, Hancock GR. The Role of measurement quality on practical guidelines for assessing measurement and structural invariance. *Educ Psychol Meas* 2016; 76: 533–561. doi: 10.1177/0013164415603764 29795877 PMC5965567

[71] Chen FF. Sensitivity of goodness of fit indexes to lack of measurement invariance. *Struct Equ Modeling* 2007; 14: 464–504.

[72] Nunnally JC, Bernstein I. *Psychometric theory*. 3rd ed. McGraw-Hill; New York: 1994.

[73] Lance CE, Butts MM, Michels LC. The Sources of Four Commonly Reported Cutoff Criteria: What Did They Really Say? *Organ Res Methods* 2006; 9: 202–220.

[74] Clark LA, Watson D. Constructing validity: Basic issues in objective scale development. *Psychol Assess* 1995; 7: 309–319.

[75] Steinberg L. *Adolescence 6th*. 6th Edition. Boston: McGraw-Hill, 2001.

[76] Rice KG, Lopez FG, Vergara D. Parental/social influences on perfectionism and adult attachment orientations. *J Soc Clin Psychol* 2005; 24: 580–605.

[77] Field S, Hoffman A, Posch M. Self-determination during adolescence: A developmental perspective. *Remedial Spec Educ* 1997; 18: 285–293.

[78] Marsh HW, Guo J, Parker PD, Nagengast B, Asparouhov T, Muthén B, et al. What to do when scalar invariance fails: The extended alignment method for multi-group factor analysis comparison of latent means across many groups. *Psychol Methods* 2018; 23: 524–545. doi: 10.1037/met0000113 28080078

[79] Stoeber J, Gaudreau P. The advantages of partialling perfectionistic strivings and perfectionistic concerns: Critical issues and recommendations. *Pers Individ Diff* 2017; 104: 379–386.

[80] Flett GL, Hewitt PL. Positive versus negative perfectionism in psychopathology: A comment on Slade and Owens’s dual process model. *Behav Modif* 2006; 30: 472–495. doi: 10.1177/0145445506288026 16723426

[81] Closson LM, Boutilier RR. Perfectionism, academic engagement, and procrastination among undergraduates: The moderating role of honors student status. *Learn Individ Differ* 2017; 57: 157–162

[82] Brown TA. *Confirmatory factor analysis for applied research* (2nd ed.). Guilford Press, 2015

[83] Kline RB. *Principles and practice of structural equation modeling* (4th ed.). Guilford Press, 2016.

[84] Asparouhov T, Muthén B. Exploratory structural equation modeling. *Struc Equ Model* 2009; 16: 397–438.

